# Twelve tips for integrating Virtual Reality Simulation into Health Professions Curricula

**DOI:** 10.12688/mep.20697.2

**Published:** 2024-11-19

**Authors:** Yvonne Finn, Siobhan Smyth

**Affiliations:** 1 School of Medicine, College of Medicine, Nursing & Health Sciences, University of Galway, Galway, H91 TK33, Ireland; 2 School of Nursing and Midwifery, College of Medicine, Nursing & Health Sciences, University of Galway, Galway, H91 TK33, Ireland

**Keywords:** Virtual Reality, VR simulation, Health professions, Simulation, Curriculum development

## Abstract

Virtual reality simulation (VRS) has the potential to disrupt and transform current understandings and practices in simulation-based education in health professions curricula. Recent technological developments, including AI applications, give the learner high levels of immersion into a virtual environment that even more closely mimic the real world than heretofore. At the same time, there are significant obstacles facing educators who strive to integrate VRS into their simulation curricula. We have written a VRS handbook for nurse educators, developed VR scenarios and delivered VRS workshops to undergraduate nursing students. Our twelve tips are aimed at undergraduate curriculum developers and simulation leaders, guiding them on how to support educators in integrating VRS into their curricula. The tips describe key considerations to be addressed in the development and integration of VRS into curricula. The tips are timely, as health professions education is on the cusp of entering technology-enhanced simulation, of which VRS will be a key player.

## Introduction

Experiential learning is embedded in modern competency-based health profession curricula, the hallmark of which is the immersion of the learner “in an experience”, followed by “reflection about the experience to develop new skills, new attitudes, or new ways of thinking” ( Lewis & Williams, 1994, p.5). Simulation-based education facilitates experiential learning, supporting the learner in acquiring essential competencies required for safe and effective patient care in their future clinical practice. A core component of simulation-based education is the scenario or patient story, which mimics the clinical and social circumstances of patients that the learner will encounter in future clinical practice. There are challenges in delivering simulation-based education, however; these include infrastructure, timetabling, availability of suitably qualified staff and costs of equipment and consumables. At the same time, the number of learners enrolled in health profession programmes has increased globally, with the number of medical students doubled and the number of nursing students increased by a third in the last decade ( Frenk *et al.*, 2022). Virtual reality simulation (VRS), defined as simulation that uses “a variety of immersive, highly visual, 3D characteristics to replicate real-life situations and/or health care procedures” can complement physical simulation and overcome some of the barriers in delivering simulation-based education to all learners during their training ( Lioce *et al.*, 2020, p.56). The advantages of VRS include flexibility in time and location, lower demand on resources and repeatability.

With the increasing growth of VRS, it is important for educators to have guidance and support to use this technology. The following twelve tips describe measures curriculum developers and simulation programme leaders can take to support health profession educators to integrate VRS into their undergraduate clinical skills and simulation curricula.

### Tip 1 Define educational theories

***Define educational theories for VRS that are aligned to the existing curriculum.*** Curriculum developers need to guide educators on educational theories they can use to inform the development of VRS, taking into account overall curriculum design and educational context. The educational theory will inform the design, content and delivery, as well as the selection of VR scenarios to deliver VRS. Kolb’s experiential learning ( Kolb, 1984), Gibbs’ reflective cycle learning theory ( Gibbs, 1988), situativity cognitive learning theory of Durning and Artino ( Durning & Artino, 2011), and McCarthy’s 4MAT learning system ( McCarthy, 1980) are some of the educational theories that can inform VRS in health profession education. Adapting an educational theory from the start will drive the educational quality of VRS.

### Tip 2 Prioritise clinical competencies for VRS

***Identify desired competencies VRS should address.*** Curriculum developers should identify which competencies their learners need more training and practice, such that they can achieve the predetermined competence level to transition to the next level of training or to graduate from the curriculum. The core competencies addressed in clinical simulation are technical skills, such as critical thinking, clinical decision-making and patient assessment, non-technical skills, such as communicating, teamwork and situation awareness, and procedural skills, such as urinary catheter insertion and NG tube insertion. Due to the complex, busy and ‘messy’ environment of health services, learners may not have achieved certain competencies to the required level of acquisition while on clinical placements. VRS provides additional experiential learning, offering opportunities for repeated practice and feedback, key factors for effective learning in simulations ( Issenberg *et al.*, 2005; Jallad & Isik, 2022). Due consideration to risk of learner fatigue and adverse effects on learners associated with longer durations of immersion in VRS e.g. nausea and dizziness, should guide decisions on the combination of, and, number of competencies to include in VR scenarios.

Curriculum developers can identify competency-training gaps through various methods, such as analysis of assessment results, learner, simulated person and educator evaluations, and focus groups or interviews with learners, educators and clinical staff. This can then inform key competencies that should be included in the VRS aspects of the curriculum.

### Tip 3 Engage key supporting staff

***Engage simulation technicians and learning technologists in VRS.*** Key support staff for VRS are simulation technicians and learning technologists. Simulation technicians have responsibility for space set-up, checking headsets, hand controllers, PCs/laptops and other necessary hardware, *‘booting up’* the VR software and doing initial checks, running the VR scenario software and being available in case of technical glitches during a VRS. Other duties include health and safety checks, maintenance, and cleaning and storage of VR hardware. Training of simulation technicians in the necessary skills to competently fulfil these duties is key to the success of implementing VRS into health professions curricula.

Curriculum developers also have access to learning technologists, who the Association for Learning Technology define as “people who are actively involved in managing, researching, supporting or enabling learning with the use of learning technology” ( https://www.alt.ac.uk). Learning technologists can champion VRS and run seminars and workshops for curriculum developers, educators and technicians. They can also take on a supervisory role in the initial introduction of VRS into health profession curricula. Finally, they can support educators as they develop teaching skills and expertise in VRS.

### Tip 4 Training of educators in VRS

***Facilitate training of educators to enable them to integrate VRS into their teaching.*** Educators need guidance and training in VRS to enable them to incorporate VRS into their teaching. An educational framework can guide and inform integration of VRS into clinical modules. The ViReTrain project developed an educational module structure, derived from a pedagogical framework, which can be used and adapted by educators in nursing education ( Smyth *et al.*, 2023). This module structure contains three core elements, namely, learning activities, simulation-based training (which includes VRS) and evaluation. A set of competencies inform the learning outcomes, which in turn are aligned to the learning activities, simulation-based training and evaluation activities of the module. Examples of learning activities are didactic lectures, group-work and case-based learning. Simulation-based training can include low-fidelity and high-fidelity simulation, and VRS. Evaluation activities can be in the form of reflective workshops, questionnaires and pre- and post-assessments.

Like physical simulation, VRS should have a prebrief phase before, and a debriefing phase after the VRS. The purpose of prebriefing is to establish a psychologically safe environment to optimize learner engagement and acquisition of skills; debriefing is a reflective process that takes place immediately after VRS, led by an educator who facilitates the process with the aid of evidence-based debriefing models and related tools. Examples include the 3D Model of Debriefing ( Zigmont *et al.*, 2011), PEARLS Healthcare Debriefing tool ( Eppich & Cheng, 2015) and Plus-Delta Debriefing ( Cheng *et al.*, 2021). There is insufficient evidence currently on the ideal functions of the educator during the VRS but most experts in VRS agree that the educator has a supervisory role during the VRS. The educator should be given control to pause or end the VRS at any time, should that be in the best interest of the learner’s safety and their learning. Leaders in healthcare simulation, such as Harvard Center for Medical Simulation ( https://harvardmedsim.org) and CAN-Sim ( https://can-sim.ca) offer a range of courses in simulation instruction.

### Tip 5 Educational materials for VRS

***Create a repository of educational materials to guide the integration of VRS into health profession curricula.*** With support from learning technologists and simulation programme leaders, curriculum developers should set up a VRS repository of educational materials. Using modern information technology (IT) and multimedia, resources can be accessed by educators at a time and place of their choosing. Educational materials can be in written, audio, video and even in VR software formats, sourced from formal websites, scientific journal databases, social media platforms, blogs and apps.

Leading healthcare education simulation organizations provide useful resources, which can be linked to local repositories. These include the International Nursing Association for Clinical Simulation and Learning ( https://www.inacsl.org/), the Association for Simulation Practice in Healthcare ( https://aspih.org.uk/) and the Society for Simulation in Healthcare ( https://www.ssih.org/). These organisations also publish standards for simulation in healthcare education, which include VRS.

### Tip 6 Source high-quality VR scenarios

***Help source high-quality VR scenarios that meet curriculum requirements.*** The quality of VR scenarios should be high, in terms of their design being informed by educational theories and frameworks, provision of a high level of immersion and being aligned to the intended competencies of the VRS. High levels of immersion are achieved when the learner perceives they are physically and mentally immersed in the VR environment. There is an emerging evidence base that high-immersion VRS is effective in the acquisition of cognitive and psychomotor competencies ( Pernica *et al.*, 2023).

VR scenarios can be purchased from commercial VR companies, or, more recently, VR scenarios can be sourced that have been created by academic institutions in collaboration with software developers. Whatever the source, curriculum developers and simulation leaders should aim to use VR scenarios that are of high quality and meet their curricular needs.

An alternative or complementary way of securing high-quality VR scenarios is to support educators to develop scenarios in collaboration with a software partner. If this path is chosen, a health simulation authoring platform, such as iRIS ( https://www.irissimulationauthoring.com), whose templates meet international standards of best practice in simulation scenario development, is highly recommended. iRIS also gives educators access to a shared library of scenarios, which includes VR scenarios.

Finally, consideration should be given to designing a curriculum with VR scenarios, which are of increasing complexity as the learner advances from the first year to the final year of their undergraduate training.

### Tip 7 Identify suitable VR hardware

***Source VR hardware that gives the learner high-immersion VR experiences.*** Examples of manufacturers of VR hardware are Meta ( www.meta.com), STEAM VR ( https://store.steampowered.com/steamvr), Apple ( www.apple.com) and Zhuoyuan ( www.funinvr.com). VR hardware include headsets, hand-controllers and some models have base stations. Headsets have cameras, sensors, headphones and speakers, and can be adjusted to fit the head size of the learner. Designs vary and this can impact comfort, weight and cleansing processes. Some headsets require cable connections to electricity supply and/or computers during use. Hand controllers also have different designs and functionality. Hand controllers allow the learners to interact in the virtual environment; hands-free interactions are also possible, depending on the software in use. Manufacturers offer advice on using their VR hardware and provide training VR software on the use, functionality, cleaning and storage of their VR hardware to clients. The cost of VR kits varies between manufacturers and the latest versions of VR kits are more expensive than earlier versions. It is recommended that educators are given opportunities to trial and compare different VR hardware before a decision is made on which model best meets their needs. Finally, educators should know that, frequently, VR software is designed to work with a particular manufacturer’s VR hardware. This may also be limited to compatibility with certain VR products from the same manufacturer. Software developers sometimes cease to offer or support VR software, putting pressure on educators to buy their latest software and newer VR hardware products. Co-design of VR software between educators and software developers may protect educators from these market pressures, as a contract can be put in place where the software developer agrees to offer and maintain the VR software for a number of years.

Finally, where educators wish to ‘cast’ the VRS onto screens, a computer is needed to run the VR scenario software.

### Tip 8 Health and safety policy for VRS

***Implement a health and safety policy for VRS.*** Educators should be supported in drawing up a health and safety policy. VRS policies should cover learner eligibility, practices to minimise adverse reactions, space requirements, and cleaning and storage of VR equipment. Learners can be assessed using an eligibility assessment tool or a user declaration form. The ViReTrain group adapted an eligibility assessment tool, developed by Southgate *et al.* (2017) ( Smyth *et al.*, 2023, section 1.4).

Minimum space requirements should be provided for VRS, avoiding close proximity to windows, doors or stairs, and keeping the space free from furniture and equipment.

A ‘spotter’, who can ensure that the learner is safe in the physical environment during the VRS, should always be in place. Breaks can be built into the VRS, particularly when the learner verbalises signs of discomfort or the educator notes signs of discomfort. Some VR software allows the learner to be seated, which can reduce the risk of dizziness and nausea. The health and safety policy should include cleaning procedures for VR hardware; this can include cleaning wipes, soaps, cleaning solutions, and clean rooms. VRS health and safety policies should be reviewed regularly and updated as required. 

### Tip 9 Evaluation of VRS

***Develop an evaluation strategy for VRS.*** Curriculum developers and simulation programme leaders should assist educators in developing an evaluation strategy for VRS. Specific evaluation tools exist to evaluate the usability of VRS, such as the System Usability Scale ( Brooke, 1986). Other tools measure learner perceptions and satisfaction; examples are the Student Perception of Effective Teaching in Simulation Scale and the Educational Satisfaction Scale. The Debriefing Assessment for Simulation in Healthcare tool evaluates strategies and techniques used by educators in the debriefing. The International Nursing Association of Clinical Simulation and Learning (INACSL) has a comprehensive repository of evaluation instruments on its website ( https://www.inacsl.org/repository-of-instruments). Other measures of evaluation can include assessment results, and the use of surveys and focus groups. It is strongly recommended that the creation of an evaluation strategy is a collaborative process with the involvement of key stakeholders, such as technical support staff, educators and students, and follows principles and practices of best practice in the evaluation of health profession education ( O’Sullivan, 2005).

### Tip 10 Networking across disciplines and institutions

***Support educators in engaging in a range of networking activities.*** By supporting networking activities, such as attendance at conferences, seminars and webinars, and through membership activities of relevant international health professions education organisations, curriculum developers can encourage collaborations in VRS, across disciplines, institutions and geographical regions. This will provide opportunities to connect with educators, learn about different approaches, barriers and facilitators to this relatively new teaching method, share innovations in VRS and co-create VR scenarios.

### Tip 11 Support research in VRS

***Advance research in VRS in health professions education.*** Research in health professions education is essential to advance knowledge and innovations, especially in emerging new teaching and learning methods, such as VRS. This can support improvements in the design and delivery of VRS, which can lead to the development of high-quality VR scenarios and high-immersion VRS in health professions curricula. Research in VRS is limited at this time, with reports of limited studies, low numbers of participants and conflicting results ( Chen *et al.*, 2020; Steen *et al.*, 2024). This adds to the call on curriculum developers to support educators to engage in VRS research.

### Tip 12 Resources/Budget allocation

***Review simulation budgets to include VRS.*** VRS offers experiential learning with the advantage of lower running costs than physical simulation. Therefore, integration of VRS into simulation programmes can reduce dependency on physical simulation alone and reduce overall costs, leaving some budget to fund VRS initiatives in the curriculum. Consequently, an increase in simulation programme budgets is not essential to fund the integration of VRS into health profession education. At the same time, the provision of additional funding to support scholarly activities, such as VR scenario development and research in VRS, is highly recommended.

## Conclusions

There is an increasing recognition that VRS complements physical simulation and, with rapid developments in VR and AI technologies, VRS can offer experiential learning opportunities for learners that closely mimic the real world of clinical practice. To overcome barriers, such as IT issues and lack of expertise with VRS, these 12 tips can guide curriculum developers and simulation leaders on how best they can support their educators to integrate high-quality, high-immersion VRS into their curricula. 

## Ethics and consent

Ethical approval and consent were not required.

## Data Availability

No data are associated with this paper.
