# Supplementary material for: Twelve tips for integrating Virtual Reality Simulation into Health Professions Curricula
Source: MedEdPublish (2016). 2024 Nov 19;14:233. Originally published 2024 Oct 16. [Version 2] doi: 10.12688/mep.20697.2 (PMC11568370; doi:10.12688/mep.20697.2)
Supplement: Peer review history

# Reviewer report: Reviewer response for version 2

**DOI:** 10.21956/mep.22279.r40165

**Published:** 2024-12-03

**Authors:** Luciana Mara Monti Fonseca

**Affiliations:** 1 Universidade de Sao Paulo, São Paulo, State of São Paulo, Brazil

The manuscript is appropriate, current, and relevant to the field. It discusses the integration of virtual reality simulation (VRS) in healthcare professional education, highlighting its transformative potential for teaching in areas such as nursing. The manuscript mentions the practical application of the tips in workshops and curriculum development, but it does not provide specific data or sufficient details for a complete reproduction of the described activities. The use of examples could be more detailed.

If evidence from practice is presented, are all the underlying source data available to ensure full reproducibility?

Partly

Is the topic of the practical tips discussed accurately in the context of the current literature

Yes

Are all factual statements correct and adequately supported by citations?

Yes

Are the conclusions drawn balanced and justified on the basis of the presented arguments?

Yes

Are arguments sufficiently supported by evidence from the published literature and/or the authors’ practice?

Yes

Reviewer Expertise:

Nursing education and Virtual simulation

I confirm that I have read this submission and believe that I have an appropriate level of expertise to confirm that it is of an acceptable scientific standard.

# Reviewer report: Reviewer response for version 2

**DOI:** 10.21956/mep.22279.r39995

**Published:** 2024-11-30

**Authors:** Melanie Barlow

**Affiliations:** 1 Australian Catholic University, Fitzroy, Victoria, Australia

It is a worthwhile topic as many higher education organizations are trying to work out how to implement VR into curriculum.

 VR is often used as standalone deliberate practice where the feedback is automated in the activity. This is an important element in the scaffolding of learning and where VR can really support independent deliberate practice to enhance skill development. It appears from the paper that VR is being used only when a facilitator is present e.g., Tips 2& 4. Are the authors advocating for VR only to be used as a facilitated activity? I think it depends on learning objectives and level of learner. Maybe some clarification around this - does debriefing still occur if students have practiced at home for example and then come to class?

If evidence from practice is presented, are all the underlying source data available to ensure full reproducibility?

Not applicable

Is the topic of the practical tips discussed accurately in the context of the current literature

Yes

Are all factual statements correct and adequately supported by citations?

Yes

Are the conclusions drawn balanced and justified on the basis of the presented arguments?

Yes

Are arguments sufficiently supported by evidence from the published literature and/or the authors’ practice?

Yes

Reviewer Expertise:

Simulation, education - clincial and  higher education

I confirm that I have read this submission and believe that I have an appropriate level of expertise to confirm that it is of an acceptable scientific standard.

# Reviewer report: Reviewer response for version 2

**DOI:** 10.21956/mep.22279.r40149

**Published:** 2024-11-20

**Authors:** Tanisha Jowsey

**Affiliations:** 1 Bond University, Robina, Queensland, Australia

The paper is improved and reads well, thanks and best wishes for this.

If evidence from practice is presented, are all the underlying source data available to ensure full reproducibility?

Yes

Is the topic of the practical tips discussed accurately in the context of the current literature

Yes

Are all factual statements correct and adequately supported by citations?

Yes

Are the conclusions drawn balanced and justified on the basis of the presented arguments?

Yes

Are arguments sufficiently supported by evidence from the published literature and/or the authors’ practice?

Yes

Reviewer Expertise:

I have ten years of teaching and research experience with healthcare simulation, including VR. I have previously taught clinical educators how to run simulation training and assessments. In my current role, I teach into a medical program and conduct medical education research.

I confirm that I have read this submission and believe that I have an appropriate level of expertise to confirm that it is of an acceptable scientific standard.

# Reviewer report: Reviewer response for version 1

**DOI:** 10.21956/mep.22157.r39890

**Published:** 2024-11-15

**Authors:** Elisabeth Coyne

**Affiliations:** 1 Griffith University, Brisbane, Australia

This is a well written article providing a simple format to present the 12 tips. The background provides an explanation of virtual simulation and highlights the significance of ensuring VRS is set up correctly within curriculum and educators understand the process and educational aspects of VRS. 

 Each tip summarises one aspect required to ensure VRS is presented to students with underlying theory and clear aims for learning. Tip 1 presents the idea of ensuring that educational theories underpin and align with the VRS. A range of theories could have been presented as providing just one may influence readers. 

 In tip 2 the prioritise clinical competency could have also highlighted the need to review the time aspect of VRS to ensure students are not overloaded with concepts within a short period or expected to be emersed in VRS for a longer period.  VRS works well as a short emersion to ensure students don’t become fatigued by the situation. 

 Tip 7 and 8 are very important and may be better as an earlier tip to ensure the empathise on student and educator health and safety is not lost as educators become excited with the newer tools of VRS.  Educator training and student debrief are major factors of VRS and need to be supported to ensure a good educational experience. 

 The idea of a repository and sharing or VRS or networking is very important to enable a range of environments with less need for individual development.

If evidence from practice is presented, are all the underlying source data available to ensure full reproducibility?

Yes

Is the topic of the practical tips discussed accurately in the context of the current literature

Yes

Are all factual statements correct and adequately supported by citations?

Partly

Are the conclusions drawn balanced and justified on the basis of the presented arguments?

Yes

Are arguments sufficiently supported by evidence from the published literature and/or the authors’ practice?

Partly

Reviewer Expertise:

digital health virtual reality

I confirm that I have read this submission and believe that I have an appropriate level of expertise to confirm that it is of an acceptable scientific standard.

## References

[1] : Simulation videos presented in a blended learning platform to improve Australian nursing students' knowledge of family assessment. https://doi.org/10.1016/j.nedt.2018.04.01285 10.1016/j.nedt.2018.04.01285 29689461

[2] : A review of virtual-simulation for assessing healthcare students' clinical competency. https://doi.org/10.1016/j.nedt.2020.104623 10.1016/j.nedt.2020.104623 33125979

# Response

**Published:** 2024-11-17

**Authors:** Yvonne Finn

**Affiliations:** School of Medicine, University of Galway, Galway, County Galway, Ireland

Dear Elisabeth,

 Many thanks for reviewing our article *Twelve tips for integrating Virtual Reality Simulation into Health Professions Currciula* and for your valuable feedback.

 I agree presentation of a range of educational theories in Tip 1 will strengthen the information provided for readers and will update this tip accordingly.

 Your comment on Tip 2, highlights the need to achieve balance between addressing prioritised clinical competencies identified with avoidance of overloading the learner and learner fatigue.

 I will update the article to bring this valuable point into Tip 2. 

 Once again, many thanks for your feedback.

# Reviewer report: Reviewer response for version 1

**DOI:** 10.21956/mep.22157.r39895

**Published:** 2024-11-12

**Authors:** Tanisha Jowsey

**Affiliations:** 1 Bond University, Robina, Queensland, Australia

Twelve tips is a great idea. It offers a digestible format and can be especially helpful for those who are new to a space. Indeed, I suspect this is where your audience will be. And virtual reality offers a rich space for learning across the health professions. I read this paper with interest. With your readers in mind, I offer these suggestions:

 Tip 1: "Kolb’s experiential learning (Kolb, 1984), constructivism, situated learning and social constructivism are some educational theories that can inform VRS in health profession education." - this could potentially be more helpful if the authors listed and referenced five or six other key theorists. Presently, Kolb is the only person referenced here and i found myself wondering, why Kolb? Why not Gibbs, Yardley, or McCarthy? It would be helpful to include reference to seminal works that use situated learning, constructivism, and social constructivism. Otherwise the reader is at risk of thinking that Kolb is the only person worth reading up on.

 Tip 2: "The core competencies addressed in clinical simulation are technical skills, such as critical thinking, clinical decision-making and patient assessment, and, non-technical skills, such as communicating, teamwork and situation awareness" - what about procedural skills? I expected these to get a mention, since heaps of clinical simulation - including VR - learning includes procedural skills. You can VR simulate inserting a catheter or an NG tube, for example.

 Tip 7 - it might be worth warning readers that one of the key issues educators experience with VR is that the software is wedded to the hardware and sometimes software developers simply cease to offer a software, thus making the hardware utterly useless. Rather than offering an update, they say 'buy our new fancy gadget.' This has major cost implications as well, of course, sustainability implications.

 Overall, this is a helpful paper. Thanks and best wishes for it.

If evidence from practice is presented, are all the underlying source data available to ensure full reproducibility?

Yes

Is the topic of the practical tips discussed accurately in the context of the current literature

Yes

Are all factual statements correct and adequately supported by citations?

Yes

Are the conclusions drawn balanced and justified on the basis of the presented arguments?

Yes

Are arguments sufficiently supported by evidence from the published literature and/or the authors’ practice?

Yes

Reviewer Expertise:

I have ten years of teaching and research experience with healthcare simulation, including VR. I have previously taught clinical educators how to run simulation training and assessments. In my current role, I teach into a medical program and conduct medical education research.

I confirm that I have read this submission and believe that I have an appropriate level of expertise to confirm that it is of an acceptable scientific standard.
